# GWEPS WORKING TO SUPPORT THE AGE-FRIENDLY HEALTHCARE SYSTEM MOVEMENT

**DOI:** 10.1093/geroni/igz038.1362

**Published:** 2019-11-08

**Authors:** Tara A Cortes, Cinnamon St.John, Jeff Lucas

**Affiliations:** 1 Hartford Institute for Geriatric Nursing at NYU Rory Meyers College of Nursing, New York, New York, United States; 2 Hartford Institute for Geriatric Nursing, United States of America, New York, United States; 3 RAIN, Bronx, New York, United States

## Abstract

Age friendly health systems aim to help people age and die with dignity. As this social movement progresses it is important to remember the community as a critical stakeholder. To ensure their engagement requires input from the community to understand their needs and education of the community to empower people to know what standard should be expected from an age friendly health system. The NYU GWEP has trained 140 community volunteers who have educated >4,500 older adults in the Bronx on healthy behaviors and chronic disease management. 85% responded that they have changed their behaviors as a result of the teaching. Qualitative data reveals that people feel they can now talk to their primary care provider in a meaningful way about what matters to them, the medications they take, their mental state, and mobility.

